# The New Spirit among Probationers

**Published:** 1919-08-02

**Authors:** 


### THE MATRONS' AND SISTERS' DEPARTMENT.

### THE NEW SPIRIT AMONG PROBATIONERS.

The women now entering for training are less inclined than their predecessors to be nurses and nothing else, and, within limits, this is all for the good.

That women should not drop all other interests when they enter their training-school is abundantly testified. How often one comes across a nurse of many years' standing who deeply regrets that in earlier days she had no opportunity or energy to keep up piano-playing, practise singing, or develop whatever gift or hobby she had. She realises too late of how much value it would have been to her as a sister in an institution or as private nurse. If her inclination be to work among children it will be well for her if her imagination can still open to the visions of childhood and if she can still weave stories for her little charges as years ago she may have done for young sisters and brothers. Children respond swiftly and with unswerving loyalty to those who understand them, and for such a nurse many difficulties in the path are swept away.

It is not enough for a nurse to sympathise, she must understand her patients' troubles and difficulties, or the sympathy will be of little value. Therefore the wider the nurse's interests, the greater help she is likely to be to those in her care, once the serious part of the illness which has made demands almost solely upon her technical skill is passed. It is sometimes said that a clergyman should be trained in a profession, or spend some time in a business firm before he is ordained in order that he may understand the ways of the world and so be of greater help to those who live in his parish. It is equally important that a woman who comes into such intimate contact with those in her care should have the gift of understanding. Strange and often pathetic are the tales poured into the nurse's ears; to her patients she is a being apart, and intimate troubles are told to her which, were she garbed in any but a nurse's uniform, would fail to come to her knowledge, yet how often these confidences give the clue to the patient's character, or illness, of which they are possibly a by-product. Surely, then, it is desirable that in learning the technique of the art of healing, the widening and the deepening of character should not be overlooked.

One of the pioneers of the nursing world, long since dead, said to a would-be probationer, "Nursing is a vocation, my dear, not the highest vocation for woman—that, though you may think it strange for me to say it, is marriage. It never came my way, so I took the second best, it is a very good second best, but remember, it is not the best. If the best does not come to you, try to make a first-class job of the second best." Such a woman was an inspiration to those who were trained in her nursing school, and inspiration is a crying need in the nursing world, as in many others at the present moment. Nursing is a profession, an art, a vocation. Its followers should be adequately paid, and should have leisure to "live their own lives," in other words, leisure to recuperate from the constant mental and spiritual, as well as physical, strain of their work. That there should be definite rules and regulations in the interests of the nurse as well as in the interests of the employer is no bar to good work, no impediment to making out of the second best, a first-class vocation.

